# Clinical and Biochemical Differences in Patients Having Non-Variceal Upper Gastrointestinal Bleeding on NSAIDs, Oral Anticoagulants, and Antiplatelet Therapy

**DOI:** 10.3390/jcm13185622

**Published:** 2024-09-22

**Authors:** Melania Ardelean, Roxana Buzas, Ovidiu Ardelean, Marius Preda, Stelian Ion Morariu, Codrina Mihaela Levai, Ciprian Ilie Rosca, Daniel Florin Lighezan, Nilima Rajpal Kundnani

**Affiliations:** 1 1st Medical Semiology, Internal Medicine, Department V, Center for Advanced Research in Cardiovascular Pathology and in Hemostaseology, “Victor Babes” University of Medicine and Pharmacy, 300041 Timisoara, Romaniadlighezan@umft.ro (D.F.L.); 2 Second Discipline of Surgical Semiology, Department IX—Surgery—1, “Victor Babes” University of Medicine and Pharmacy, 300041 Timisoara, Romania; 3 Second Clinic of General Surgery and Surgical Oncology, Emergency Clinical Municipal Hospital, 300079 Timisoara, Romania; 4 Breast Surgery Research Center, “Victor Babes” University of Medicine and Pharmacy, 300041 Timisoara, Romania; 5 Faculty of Medicine, University of Medicine “Vasile Goldis”, 310025 Arad, Romania; 6 Discipline of Medical Communications, Department II—Microscopic Morphology, “Victor Babes” University of Medicine and Pharmacy, 300041 Timisoara, Romania; 7 Discipline of Internal Medicine and Ambulatory Care, Prevention and Cardiovascular Recovery, Department VI—Cardiology, “Victor Babes” University of Medicine and Pharmacy, 300041 Timisoara, Romania; knilima@umft.ro; 8 Research Centre of Timisoara Institute of Cardiovascular Diseases, “Victor Babes” University of Medicine and Pharmacy, 300041 Timisoara, Romania

**Keywords:** non-variceal bleeding, UGIB, NOAC, NSAID, VKA, Rockall score

## Abstract

**Introduction:** Upper gastrointestinal bleeding (UGIB) is among the most common causes of morbidity and mortality worldwide, accounting for major resource allocation and increasing incidence. This study aimed to evaluate the severity of non-variceal bleeding in patients at risk of bleeding through the use of NSAIDs, oral anticoagulants, and antiplatelet therapy. **Material and Method:** The study included 296 patients admitted in the Gastroenterology Department of the Municipal County Emergency University Hospital, Timisoara, between 01.01.2018 and 01.04.2020, and diagnosed via gastroscopy with non-variceal gastrointestinal bleeding. The patients were divided among four groups based on their use of different drugs known to induce UGIB, i.e., aspirin and clopidogrel, NOACs, NSAIDs, and anti-vitamin K drugs, respectively. Statistical analyses were performed based on ANOVA one-way tests for continuous variables and Chi-square tests for categorical variables with pairwise comparisons based on Bonferroni adjusted significance tests. **Results:** The results showed several parameters having statistical significance among the different groups of patients. Patients on NOACs had statistically significant lower hemoglobin levels, lower hematocrit values, lower erythrocytes, lower RDW and higher fibrinogen levels compared to patients on VKA. **Discussion:** Surprisingly, the results from our study suggest that the use of NOACs was associated with a higher risk of bleeding when compared to VKA, which differs from the existing literature. **Conclusions:** One of the important factors causing upper non-variceal bleeding can be iatrogenic, either due to antiplatelet drugs or anticoagulants, to which NSAID treatment is additionally associated for various reasons. In our study, the use of NOACs seemed to have a more severe bleeding spectrum with higher morbidity compared to VKA.

## 1. Introduction

Peptic ulcers account for approximately 50% of gastrointestinal tract bleeding causes originating in the upper gastrointestinal tract [1]. Upper gastrointestinal bleeding (UGIB) carries a high mortality, especially in elderly patients having co-morbidities, and the incidence of non-variceal and variceal bleeding is reported to be 3.5% and 15%, respectively [2,3]. The incidence of upper gastrointestinal bleeding due to peptic ulcer disease is steadily decreasing and this decrease can be attributed to several factors. The use of *Helicobacter pylori* eradication therapies, the widespread use of proton pump inhibitors, and easier access to screening procedures, including gastroscopy, all contribute to varying degrees to the reduction in incidence [3,4].

However, the current incidence of UGIB in developed countries is around 60–100 cases per 100,000 inhabitants, a roughly stable trend over the last 20 years, suggesting that there are some contributing factors [5]. Atrial fibrillation, deep vein thrombosis, pulmonary embolism, and other conditions require anticoagulation and antiplatelet therapy [6,7,8] putting the patients at high risk of hemorrhage [9]. Furthermore, population aging, secondary cardiovascular disease prophylaxis involving antiplatelet therapy, increased consumption of non-steroidal anti-inflammatory drugs (NSAIDs), prolonged survival after neoplastic diseases, and prolonged institutionalization in acute and chronic neurological diseases contribute significantly to the maintenance of this clinical diagnosis in daily practice [10].

The latest AHA report on cardiovascular diseases [11] suggests, in the most optimistic scenarios, a doubling of the prevalence of atrial fibrillation by 2030 compared to 2010. The need to institute anticoagulation for this condition, both as primary and secondary prevention, will automatically increase the number of UGIB cases. An improvement in this regard has occurred with the advent of novel direct oral anticoagulants (NOACs,) which have been shown to be non-inferior to vitamin K antagonists (VKAs) [12]. However, the rise in the incidence of UGIB seems inevitable.

The use of drugs that represent a disturbance in the balance of protective factors in the gastric mucosa creates the necessary prerequisites for the appearance of UGIB. NSAIDs [13], VKAs, and NOACs [14] are directly involved in this pathophysiological process.

UGIB can be classified based on its etiology (variceal vs. non-variceal) and based on its involvement in the GI tract (esophagus, stomach, duodenum, and others). Examples of UGIB include [5,15,16,17] (1) esophageal bleeding: erosive esophagitis, esophageal varices, Mallory Weiss tear; (2) gastric bleeding: peptic ulcer, gastritis, varices or neoplasia; (3) duodenal bleeding: peptic ulcer and erosive duodenitis; and (4) Dieulafoy ulcer and angiodysplasia from different portions of the GI tract. In the event of UGIB, the Rockall score [18] is a valid prediction score, repeatedly confirmed [19] to assess the risk of patients with non-variceal bleeding, which includes parameters such as age, co-morbidities, presence of shock, diagnosed bleeding source, and stigmata of recent bleeding that can be rapidly identified; the management of UGIB is commonly adapted according to this score.

The aim of this retrospective study was to highlight the severity of the association between non-variceal UGIB and the use of NSAIDs, antiplatelets, VKAs, and NOACs (dabigatran, apixaban, rivaroxaban) in patients.

## 2. Materials and Methods

This study included 296 patients with non-variceal UGIB admitted to the Gastroenterology Department of the Municipal County Emergency University Hospital, Timisoara, between 1 January 2018 and 1 April 2020.

The patients were all older than 18 years and presented either to the ER or the outpatient department with signs and symptoms of upper GI bleeding. The data were collected by reviewing medical charts and collecting demographic information: careful history, with emphasis on recent medication history, anticoagulant treatment, antiplatelet therapy, NSAID consumption in the last 14 days, as well as the associated parameters required to calculate the Rockall score. We excluded UGIBs caused by variceal rupture or patients with hepatic cirrhosis.

Patients were divided into 4 groups. The first group included patients on aspirin and clopidogrel, group number 2 included patients on NOAC, group number 3 included patients on NSAIDs, and group number 4 included patients on antivitamin K (VKA) therapy.

All patients underwent vital parameters monitoring upon admission and ECGs were performed of every patient for the evaluation of rate and rhythm, to exclude arrhythmias, ischemia, ST elevation, and other specific findings. For the assessment of shock status, heart rate and blood pressure were measured in both arms and the highest value was considered [20,21,22].

All patients underwent gastroscopy within the first 24 h of admission and specific elements of bleeding, while recent bleeding stigmata considered relevant according to the Rockall score were noted. The Rockall score was divided according to risk classes into low-risk Rockall score (≤2 points), noted in the tables with 0, intermediate risk (3–5 points), noted in the tables with 1, and high risk (≥6 points), noted with 2 in the tables, respectively. Thus, we intended to identify high-risk patients who may benefit from longer hospitalization.

The study was approved by the Scientific Research Ethics Committee of the Municipal County Emergency University Hospital, Timisoara, Romania (approval number: I-9645 dated 26 April 2022), and was conducted in accordance with the Helsinki Declaration. Written informed consent was obtained as a part of routine procedure from all the patients admitted at our university hospitals for further research publication and educational purposes.

### Statistical Analysis

A statistical analysis was performed using IBM SPSS Statistics version 20.0 software for Windows with a significance level of 0.05. We used descriptive statistics, figures, and tables to summarize our findings.

We analyzed our patients based on the groups of patients as aforementioned. The statistical analysis was based on ANOVA one-way tests for continuous variables and Chi-square tests for categorical variables, with the corresponding *p*-values being presented in the summary tables. Pairwise comparisons were performed based on Bonferroni adjusted significance tests, with the corresponding significance (S) (i.e., *p* ≤ 0.05) or non-significance (NS) (i.e., *p* > 0.05) being reported. Our primary outcome was to determine whether there was a statistically significant difference in the clinical parameters that indicate morbidity and bleeding risks such as hemoglobin, hematocrit, RDW, RBCs, thrombocytes, leucocytes, creatinine, fibrinogen, liver enzymes, ulcers, cancer, atrial fibrillation, coronary artery disease, and diabetes mellitus upon comparing the 4 groups of patients. We also incorporated descriptive statistics.

Results for targeted variables are presented using descriptive statistics (mean, standard deviation, range, median, interquartile range) for continuous data, and counts with associated percentages for categorical data. We analyzed our patients based on the four groups of patients by comparing independently groups 1, 2, and 3 to group 4. An independent samples *t*-test was used to analyze differences in means for continuous variables, while differences between categorical variables were examined by a Chi-squared test. *p*-Values based on the Bonferroni correction from the comparison between group 1 and group 2 patients are flagged under the *p*-value 1 column in the summary tables, *p*-value 2 flag the comparison result of group 1 with group 3 patients, and *p*-value 3 flag the comparison result of group 2 with group 3 patients.

## 3. Results

Table 1 shows baseline characteristics of the data with mean (with standard deviation) and median (with Q1 and Q3). Table 2 and Table 3 provides an overall summary of the main characteristics such as age, gender, heart rate, BP, Rockall score, etc., followed by the laboratory test results and clinical characteristics for each group.

Our data analysis (Table 3) suggested the following statistically significant findings.

Analysis of patients on NOACS vs. those on VKAs:

- Group 2 patients (on NOACs) had lower hemoglobin levels compared to group 4 patients (on VKA) (6.408 vs. 8.065, statistically significant with *p* = 0.027).
- Group 2 patients had lower hematocrit values compared to group 4 patients (20.175 vs. 25.850, *p* = 0.014).
- Group 2 patients had a lower erythrocyte count compared to group 4 patients (2.254 vs. 2.994, *p* = 0.005).
- Group 2 patients had smaller RDWs compared to group 4 patients (14.667 vs. 16.688, *p* = 0.070).
- Group 2 patients had higher fibrinogen levels compared to group 4 patients (5.000 vs. 3.735, *p* < 0.001).
- Group 2 patients had higher AST levels compared to group 4 patients (50.83 vs. 34.77, *p* = 0.030).
- A significantly lower proportion of patients with atrial fibrillation were in group 2 as compared to group 4 (0% vs. 39.13%, *p* = 0.007).
- Analysis of patients on NSAIDs vs. those on VKAs:
- Patients using NSAIDs were younger as compared to those using VKAs; there were more males than females in the NSAID group and more females than males in the VKA group.
- Group 3 patients (on NSAIDs) had a higher erythrocyte count compared to group 4 patients (4.131 vs. 2.994, *p* = 0.008).
- Group 3 patients had a higher thrombocyte count compared to group 4 patients (370.00 vs. 235.500, *p* < 0.001).
- Group 3 patients had larger RDWs and higher ALT levels compared to group 4 patients (19.411 vs. 16.688, *p* = 0.005, and 29.37 vs. 21.17, *p* = 0.004, respectively).
- A significantly lower proportion of patients with atrial fibrillation were in group 3 as compared to group 4 patients (15.79% vs. 39.13%, *p* = 0.052).
- A significantly lower proportion of patients with coronary artery disease were in group 3 as compared to group 4 patients (15.79% vs. 59.78%, *p* < 0.001).
- Analysis of patients on aspirin and clopidogrel vs. those on VKAs:
- Group 1 patients (on aspirin and clopidogrel) had lower thrombocyte count, compared to group 4 patients (181.39 vs. 235.50, statistically significant with *p* = 0.017).
- Group 1 patients had smaller RDWs compared to group 4 patients (13.564 vs. 16.688, *p* < 0.001).
- Group 1 patients had higher AST and ALT levels compared to group 4 patients (114.64 vs. 34.77, *p* = 0.048, and 86.46 vs. 21.17, *p* = 0.020, respectively).
- A significantly higher proportion of patients with ulcers were in group 1 as compared to group 4 patients (57.14% vs. 36.96%, *p* = 0.058).
- A significantly lower proportion of patients with diabetes mellitus were in group 1 as compared to group 4 patients (17.86% vs. 38.04%, *p* = 0.047).

## 4. Discussion

The basic characteristics are summarized in Table 1 and the results of the different comparisons are detailed in Table 2 and Table 3.

Concerning patients who were on NSAIDs, it was observed that patients experiencing bleeding and consuming NSAIDs were younger compared to treated with VKAs. A study of 86 patients with non-variceal bleeding found that 14.6% of patients had used NSAIDs and 51% had an anticoagulant treatment, with a mean age similar to our group 4 patients, i.e., 66.3 years old [23], and a much higher proportion of males in the VKA group compared to the NSAID group and no significant differences between the other groups compared. Bozkurt et al. [24] demonstrated a 3:1 male versus female ratio in a group of patients with non-variceal bleeding staged according to the Rockall score, while in our study, there were slightly more females in the VKA group.

In terms of hemodynamic status, patients in groups 2 and 3 had significant differences when compared to those on VKAs, with NOAC patients having an average systolic blood pressure 26.56 mmHg lower and those on NSAIDs having a systolic blood pressure approximately 10 mmHg lower than those on VKAs. In a univariate analysis [25], systolic blood pressure less than 90 mmHg was a significant parameter. This may suggest that patients with NOACs or NSAIDs in their recent history may have more severe bleeding, which may also be suggested by the evidence of tachycardia in NOAC patients compared to VKA patients, again with statistical significance.

In our study, there was a significantly lower proportion of patients with atrial fibrillation in the group of patients on NOACs as compared to the group of patients on VKAs (0% vs. 39.13%, *p* = 0.007). We also found that patients on NOACs had higher AST levels compared to patients on VKAs (50.83 vs. 34.77, *p* = 0.030). AST (aspartate aminotransferase) liver enzyme plays a role in amino acid metabolism since it catalyzes the reversible transfer of an alpha amino group between aspartate and glutamate. Raised AST levels are found in liver cirrhosis, viral hepatitis, and toxic liver damage. Raised AST levels signify liver dysfunction, which in turn contributes to bleeding tendency through several mechanisms [26] such as impaired synthesis of clotting factors including prothrombin and fibrinogen, vitamin K deficiency, a possibility of impending disseminated intravascular coagulation (DIC), thrombocytopenia, and endothelial dysfunction leading to impaired blood vessel integrity.

Patients on NOACs in terms of laboratory parameters had all erythrocyte indices (hemoglobin, hematocrit, erythrocytes) being lower compared to patients on VKAs. We were surprised to find that there was a higher risk of bleeding in patients on NOACs than in patients on VKA as signified by lower hemoglobin, hematocrit, and erythrocyte counts. This is the most striking finding in our study and is contrary to most articles and studies available in the current literature, since it establishes that patients on NOACs have a higher bleeding risk than patients on VKAs. Interestingly, we found a study that partially supported our finding [27]. The authors concluded that NOACs did not alter the outcomes of UGIB as compared to VKAs and were not superior to VKAs in this way.

However, group 2 patients had higher fibrinogen levels compared to group 4 patients (5.000 vs. 3.735, *p* < 0.001). Fibrinogen is produced by the liver and converted into fibrin in the coagulation cascade. It has an important role in hemostasis by various mechanisms [28]: fibrinogen binds to platelets to facilitate their aggregation, and it converts to fibrin, which forms a clot which is further stabilized by fibrinogen. Hypofibrinogenemia which is found in cirrhosis and impaired liver function, inherited fibrinogen disorders, consumptive coagulopathy of DIC and trauma/surgery, sepsis, cancer or bone marrow disorders, medications or nutritional deficiencies, pregnancy complications, and certain snake venoms, is known to cause prolonged bleeding. Hyperfibrinogenemia increases the risk of thrombosis. Lower fibrinogen levels in the VKA group suggests that patients on VKAs may be at a high overall risk of bleeding because of low fibrinogen levels, as found in the existing literature [29,30,31].

Most of the patients evaluated had a Rockall score >1. Thus, most patients had some risk of UGIB to begin with, which was then aggravated with the use of the medications studied. This limits the comparison of bleeding risk in each group of patients with a zero score. Most studies in the existing literature suggest that there is a higher risk of bleeding in patients on VKAs as compared to those on NOACs, but the patients studied in such studies had a Rockall score of zero.

Hemoglobin level is considered in some scores as an important parameter either for mortality or for re-bleeding [32]. NOAC patients in this study had a mean Hb of 6 g/dL. In terms of platelet count, patients on antiplatelet medicines had a lower platelet count compared to patients on VKAs, but patients on NSAIDs had a higher platelet count suggesting a more intense active bleeding.

Regarding the RDW, patients on antiplatelets and NOACs had a lower index, while patients on NSAIDs had a higher RDW compared to those on VKAs. The RDW is considered by some authors as a bleeding marker that could enter as a new parameter in the evaluation of these patients with non-variceal bleeding [33,34], a percentage >14.5% suggesting increased bleeding risk. In the present study, patients with NSAIDs had the highest RDW. The RDW is a useful index, as a measure of the variation in size of RBCs in the blood. The baseline RDW value is linked with long term adverse events in conditions like myocardial infarction, heart failure, angina, stroke, and peripheral artery disease [35,36,37]. Elevated RDW levels have been linked to an increased risk of bleeding tendency and mortality due to several mechanisms [38,39,40]: A high RDW is associated with altered platelet function leading to decreased platelet aggregation, inflammation, oxidative stress, and endothelial dysfunction. An elevated RDW is somewhat associated to blood coagulation disorders, such as thrombocytopenia and coagulopathy, vascular dysfunction, leading to impaired blood vessel integrity and increased bleeding risk and Von Willebrand disease [38]. Along with RDW other factors such as platelet count, coagulation studies, and clinical history should also be considered for a comprehensive evaluation for bleeding tendency [39].

Liver function in these patients revealed differences that can be considered part of the spectrum of decompensation secondary to bleeding, with patients on antiplatelets having both AST and ALT levels higher than those on VKAs, while patients on NOACs had only higher AST levels.

Patients with diabetes had a higher rate of antiplatelet prescribing probably secondary to the accelerated rate of atherosclerosis, while the number patients with atrial fibrillation was statistically significantly larger due to anticoagulation on either VKA or NOAC, respectively.

This is among the very few studies available in the literature that simultaneously compares the bleeding risk in patients on aspirin/clopidogrel, NOACs, NSAIDs and VKAs. Limitations of the study include its single-centric data and retrospective nature. We understand that the small number of patients, especially in the NOAC group, is a limitation of our study; however, as one of the first studies on this subject at our center, it is a base for further studies with hundreds of cases over several years. More studies can be implemented of this kind at multiple centers to bring strong and statistically significant findings that would further suggest the right choice of anticoagulants in different patients. Moreover, as a limitation, we did not compare the outcomes among different individual NOAC medications (e.g., dabigatran, apixaban, rivaroxaban versus each other) and individual NSAID medications used by these patients. Also, perhaps another study can be conducted to compare the results in patients on dual versus single antiplatelet therapy, and there, patients with a history of ticagrelor use can also be studied. The etiology as well as type of ulcers can be compared with various parameters in future studies.

## 5. Conclusions

The spectrum of patients with upper non-variceal bleeding is heterogeneous, with an important iatrogenic component either due to antiplatelet drugs or anticoagulants, to which NSAID treatment is additionally associated for various reasons. In the present situation, the use of NSAIDs and NOACs seemed to have a more severe bleeding spectrum compared to VKAs, as suggested by lower hemoglobin, hematocrit, and erythrocyte counts. But for this aspect, it should be mentioned that most of the patients evaluated had a Rockall score >1, limiting the power of comparison with those with a zero score.

## Figures and Tables

**Table 1:** Summary of baseline characteristics.

	All Patients (N = 296)
Age (years) (*)	
Mean (SD)	65.89 (13.587)
Min; max	25; 93
Median (Q1; Q3)	67.00 (57.50; 76.00)
Gender	
Female	136 (45.95%)
Male	160 (54.05%)
Systolic blood pressure (*)	
Mean (SD)	120.52 (23.408)
Min; max	60; 180
Median (Q1; Q3)	120.00 (110.00; 140.00)
Diastolic blood pressure (*)	
Mean (SD)	73.61 (12.396)
Min; max	40; 110
Median (Q1; Q3)	70.00 (70.00; 80.00)
Heart rate (bpm) (*)	
Mean (SD)	90.03 (17.490)
Min; max	64; 140
Median (Q1; Q3)	90.00 (75.00; 100.00)
Clinical outcome	
Death	16 (5.41%)
“Discharged”	276 (93.24%)
Transfer to ICU	4 (1.35%)
Rockall score	
0	13 (4.39%)
1	124 (41.89%)
2	159 (53.72%)

Continuous data (*) are summarized as mean (standard deviation); minimum and maximum values; median and associated quartiles (Q1—25th percentile; Q3—75th percentile).

**Table 2:** Summary of main characteristics by groups. Group 1: aspirin and clopidogrel; group 2: NOACs; group 3: NSAIDs; group 4: VKA.

	Group 1 vs. Group 4			Group 2 vs. Group 4			Group 3 vs. Group 4		
	Group 1 (N = 28)	Group 4 (N = 92)	*p*-Value	Group 2 (N = 12)	Group 4 (N = 92)	*p*-Value	Group 3 (N = 19)	Group 4 (N = 92)	*p*-Value
Age (years) Mean (SD) Min; max Median (Q1; Q3)	71.18 (14.163) 45; 93 70.00 (64.50; 81.00)	67.32 (12.320) 41; 88 69.50 (60.00; 76.50)	0.163	66.17 (13.803) 47; 77 75.00 (48.00; 75.50)	67.32 (12.320) 41; 88 69.50 (60.00; 76.50)	0.765	60.74 (8.425) 46; 71 64.00 (58.00; 66.50)	67.32 (12.320) 41; 88 69.50 (60.00; 76.50)	0.029
Gender Female Male	11 (39.29%) 17 (60.71%)	50 (54.35%) 42 (45.65%)	0.163	8 (66.67%) 4 (33.33%)	50 (54.35%) 42 (45.65%)	0.419	4 (21.05%) 15 (78.95%)	50 (54.35%) 42 (45.65%)	0.008
Systolic blood pressure (mmHg) Mean (SD) Min; max Median (Q1; Q3)	119.64 (29.025) 80; 175 110.00 (110.00; 130.00)	127.39 (21.129) 90; 180 130.00 (110.00; 140.00)	0.124	100.83 (9.003) 90; 110 100.00 (90.00; 110.00)	127.39 (21.129) 90; 180 130.00 (110.00; 140.00)	<0.001	117.37 (14.469) 90; 140 120.00 (110.00; 120.00)	127.39 (21.129) 90; 180 130.00 (110.00; 140.00)	0.016
Diastolic blood pressure (mmHg) Mean (SD) Min; max Median (Q1; Q3)	70.00 (13.053) 50; 110 70.00 (60.00; 80.00)	75.60 (12.620) 50; 105 70.00 (70.00; 80.00)	0.044	70.42 (8.649) 60; 80 70.00 (60.00; 80.00)	75.60 (12.620) 50; 105 70.00 (70.00; 80.00)	0.171	72.63 (5.367) 60; 80 70.00 (70.00; 77.50)	75.60 (12.620) 50; 105 70.00 (70.00; 80.00)	0.171
Heartrate (bpm Mean (SD) Min; max Median (Q1; Q3)	87.43 (16.347) 70; 120 85.00 (72.50; 93.50)	86.40 (19.090) 64; 140 82.50 (70.00; 96.00)	0.798	100.00 (19.531) 75; 125 102.50 (77.00; 120.00)	86.40 (19.090) 64; 140 82.50 (70.00; 96.00)	0.023	91.47 (13.087) 75; 120 90.00 (84.00; 100.00)	86.40 (19.090) 64; 140 82.50 (70.00; 96.00)	0.272
Status Death Discharged Transferred to ICU	0 (0%) 28 (100%) 0 (0%)	4 (4.35%) 84 (91.30%) 4 (4.35%)	0.271	4 (33.33%) 8 (66.67%) 0 (0%)	4 (4.35%) 84 (91.30%) 4 (4.35%)	0.002	3 (15.79%) 16 (84.21%) 0 (0%)	4 (4.35%) 84 (91.30%) 4 (4.35%)	0.123
Rockall score 0 1 2	0 (0%) 22 (78.57%) 6 (21.43%)	0 (0%) 16 (17.39%) 76 (82.61%)	<0.001	0 (0%) 8 (66.67%) 4 (33.33%)	0 (0%) 16 (17.39%) 76 (82.61%)	<0.001	0 (0%) 17 (89.47%) 2 (10.53%)	0 (0%) 16 (17.39%) 76 (82.61%)	<0.001

**Table 3:** Summary of Laboratory Test Results and Clinical Characteristics by Groups. Group 1: aspirin and clopidogrel; Group 2: NOAC; Group 3: NSAIDs; Group 4: VKA.

	Group 1 vs. Group 4			Group 2 vs. Group 4			Group 3 vs. Group 4		
	Group 1 (N = 28)	Group 4 (N = 92)	*p*-Value	Group 2 (N = 12)	Group 4 (N = 92)	*p*-Value	Group 3 (N = 19)	Group 4 (N = 92)	*p*-Value
“Hemoglobin” Mean (SD) Min; max Median (Q1; Q3)	7.718 (2.4807) 4.7; 12.5 7.500 (5.200; 9.200)	8.065 (2.4848) 3.5; 13.4 7.600 (6.700; 8.700)	0.518	6.408 (1.5553) 4.9; 8.5 5.800 (5.000; 8.400)	8.065 (2.4848) 3.5; 13.4 7.600 (6.700; 8.700)	0.027	7.953 (1.6352) 4.6; 9.8 8.200 (7.600; 8.700)	8.065 (2.4848) 3.5; 13.4 7.600 (6.700; 8.700)	0.518
”Hematocrit” Mean (SD) Min; max Median (Q1; Q3)	23.961 (7.8690) 14.4; 37.9 23.100 (14.800; 30.200)	25.850 (7.6133) 12.2; 43.6 24.200 (20.900; 28.300)	0.256	20.175 (5.1223) 15.8; 27.0 17.850 (15.800; 27.000)	25.850 (7.6133) 12.2; 43.6 24.200 (20.900; 28.300)	0.014	27.211 (6.5744) 13.8; 37.2 27.900 (26.300; 31.950)	25.850 (7.6133) 12.2; 43.6 24.200 (20.900; 28.300)	0.470
“Red blood cells” Mean (SD) Min; Max Median (Q1; Q3)	2.751 (0.8918) 1.57; 4.23 2.640 (2.070; 3.590)	2.994 (0.8589) 1.06; 4.37 3.140 (2.385; 3.730)	0.196	2.254 (0.6244) 1.68; 3.07 1.980 (1.680; 3.070)	2.994 (0.8589) 1.06; 4.37 3.140 (2.385; 3.730)	0.005	4.131 (1.6236) 1.83; 6.80 3.590 (3.320; 4.480)	2.994 (0.8589) 1.06; 4.37 3.140 (2.385; 3.730)	0.008
“Thrombocytes” Mean (SD) Min; max Median (Q1; Q3)	181.39 (89.081) 49; 378 161.50 (146.50; 194.00)	235.50 (138.271) 39; 615 186.00 (138.00; 343.00)	0.017	273.17 (223.258) 62; 569 179.00 (78.50; 568.00)	235.50 (138.271) 39; 615 186.00 (138.00; 343.00)	0.579	370.00 (152.038) 189; 618 374.00 (250.00; 440.00)	235.50 (138.271) 39; 615 186.00 (138.00; 343.00)	<0.001
“RDW” Mean (SD) Min; max Median (Q1; Q3)	13.564 (1.7685) 12.2; 20.4 13.000 (12.300; 14.000)	16.688 (3.7497) 13.0; 28.3 16.150 (13.800; 17.700)	<0.001	14.667 (1.9397) 12.8; 17.2 14.000 (12.800; 17.200)	16.688 (3.7497) 13.0; 28.3 16.150 (13.800; 17.700)	0.070	19.411 (3.6463) 14.4; 23.7 17.500 (17.100; 23.100)	16.688 (3.7497) 13.0; 28.3 16.150 (13.800; 17.700)	0.005
“Leucocytes” Mean (SD) Min; max Median (Q1; Q3)	11.142 (3.6873) 7.20; 22.00 10.900 (8.340; 12.700)	11.036 (5.2640) 5.10; 37.00 9.450 (7.550; 13.700)	0.921	8.373 (2.8537) 3.98; 10.80 9.750 (4.900; 10.700)	11.036 (5.2640) 5.10; 37.00 9.450 (7.550; 13.700)	0.089	12.443 (9.4601) 5.67; 32.50 7.800 (6.800; 14.300)	11.036 (5.2640) 5.10; 37.00 9.450 (7.550; 13.700)	0.537
“Serum creatinine” Mean (SD) Min; max Median (Q1; Q3)	1.926 (1.5544) 0.70; 5.30 1.200 (0.800; 2.990)	4.091 (14.6015) 0.52; 83.00 1.200 (0.800; 1.700)	0.436	1.345 (0.6142) 0.67; 2.40 1.200 (0.750; 2.000)	4.091 (14.6015) 0.52; 83.00 1.200 (0.800; 1.700)	0.518	0.886 (0.2013) 0.63; 1.30 0.800 (0.700; 1.100)	4.091 (14.6015) 0.52; 83.00 1.200 (0.800; 1.700)	0.343
“Fibrinogen” Mean (SD) Min; max Median (Q1; Q3)	3.982 (1.0722) 3.0; 6.4 3.700 (3.400; 3.750)	3.735 (1.4899) 1.0; 6.5 3.700 (2.600; 4.700)	0.336	5.000 (0.6661) 4.5; 5.9 4.600 (4.500; 5.900)	3.735 (1.4899) 1.0; 6.5 3.700 (2.600; 4.700)	<0.001	4.268 (1.0786) 3.1; 6.1 4.400 (3.300; 4.900)	3.735 (1.4899) 1.0; 6.5 3.700 (2.600; 4.700)	0.142
“AST” Mean (SD) Min; max Median (Q1; Q3)	114.64 (203.666) 17; 627 37.00 (23.00; 54.00)	34.77 (24.204) 12; 132 26.00 (21.50; 38.00)	0.048	50.83 (20.333) 20; 76 45.00 (33.50; 76.00)	34.77 (24.204) 12; 132 26.00 (21.50; 38.00)	0.030	31.05 (12.145) 11; 44 35.00 (23.00; 41.00)	34.77 (24.204) 12; 132 26.00 (21.50; 38.00)	0.516
“ALT” Mean (SD) Min; max Median (Q1; Q3)	86.46 (140.190) 6; 421 26.00 (14.50; 81.00)	21.17 (10.660) 7; 63 18.50 (13.00; 26.50)	0.020	30.83 (16.375) 14; 52 24.00 (18.00; 52.00)	21.17 (10.660) 7; 63 18.50 (13.00; 26.50)	0.069	29.37 (13.280) 9; 59 32.00 (19.50; 36.00)	21.17 (10.660) 7; 63 18.50 (13.00; 26.50)	0.004
“Ulcer” No Yes	12 (42.86%) 16 (57.14%)	58 (63.04%) 34 (36.96%)	0.058	7 (58.33%) 5 (41.67%)	58 (63.04%) 34 (36.96%)	0.751	9 (47.37%) 10 (52.63%)	58 (63.04%) 34 (36.96%)	0.203
Cancer No Yes	25 (89.29%) 3 (10.71%)	76 (82.61%) 16 (17.39%)	0.397	8 (66.67%) 4 (33.33%)	76 (82.61%) 16 (17.39%)	0.239	15 (78.95%) 4 (21.05%)	76 (82.61%) 16 (17.39%)	0.745
“Other (incl. erosions, petechiae, Dieulafoy)” No Yes	14 (50.00%) 14 (50.00%)	33 (35.87%) 59 (64.13%)	0.180	5 (41.67%) 7 (58.33%)	33 (35.87%) 59 (64.13%)	0.755	15 (78.95%) 4 (21.05%)	33 (35.87%) 59 (64.13%)	0.001
“Atrial fibrillation” No Yes	21 (75.00%) 7 (25.00%)	56 (60.87%) 36 (39.13%)	0.172	12 (100%) 0 (0%)	56 (60.87%) 36 (39.13%)	0.007	16 (84.21%) 3 (15.79%)	56 (60.87%) 36 (39.13%)	0.052
Coronary artery disease No Yes	16 (57.14%) 12 (42.86%)	37 (40.22%) 55 (59.78%)	0.114	8 (66.67%) 4 (33.33%)	37 (40.22%) 55 (59.78%)	0.082	16 (84.21%) 3 (15.79%)	37 (40.22%) 55 (59.78%)	<0.001
“Diabetes mellitus” No Yes	23 (82.14%) 5 (17.86%)	57 (61.96%) 35 (38.04%)	0.047	8 (66.67%) 4 (33.33%)	57 (61.96%) 35 (38.04%)	>0.999	16 (84.21%) 3 (15.79%)	57 (61.96%) 35 (38.04%)	0.063

*p*-Values were obtained with independent samples *t*-tests. Continuous data are summarized as mean (standard deviation); minimum and maximum value; median and associated quartiles (Q1—25th percentile; Q3—75th percentile). Quartiles were obtained with Tukey’s method.

## Data Availability

Data will be made available on valid written requests to the corresponding authors.
